# A case of acute unilateral maxillary atelectasis following endoscopic pituitary surgery

**DOI:** 10.1016/j.ijscr.2019.12.041

**Published:** 2020-01-14

**Authors:** Joyce P.K. Ho, Darren Rom, Eugene Wong, Narinder P. Singh

**Affiliations:** a Department of Otolaryngology Head and Neck Surgery, Westmead Hospital, Sydney, Australia; b Sydney Medical School, The University of Sydney, Sydney, Australia

**Keywords:** Endoscopic surgery, Maxillary atelectasis, Pituitary surgery, Sinus surgery, Sinusitis, Transnasal surgery

## Abstract

**Introduction:**

Chronic maxillary atelectasis (CMA) is traditionally described as a rare, acquired condition of persistent and progressive antral wall collapse causing a reduction in maxillary sinus volume. In a handful of cases, CMA has been identified in patients with previously normal computed tomography (CT) imaging. However, maxillary atelectasis occurring rapidly after pituitary surgery, despite a normal pre-operative CT scan, has not previously been described in the literature.

**Presentation of case:**

A 29-year-old male presented two months post endoscopic transnasal, transsphenoidal excision of a Rathke’s cleft cyst with facial pain and pressure, unresponsive to medical treatment. CT of the paranasal sinuses at five months post-operatively demonstrated evidence of a left CMA, which was not present on pre-operative imaging. He underwent endoscopic uncinectomy and middle meatal antrostomy with complete resolution of symptoms.

**Discussion:**

To our knowledge, this is the first case in the literature describing acute development of maxillary atelectasis. While lateralisation of the middle turbinate causing ostiomeatal obstruction may lead to sinus atelectasis, it is typically a slow process and often seen in the context of chronic disease. Review of the literature demonstrates six cases of documented rapid-onset atelectasis of maxillary sinuses. Four of these cases were post-operative, with one involving direct surgery on the affected sinus.

**Conclusion:**

This case represents the first in the literature of acute, post-operative unilateral maxillary atelectasis following pituitary surgery, where the affected sinus was not directly involved in the initial operation. Consideration should be made to incorporate acute cases into the current classification system.

## Highlights

• Maxillary atelectasis has traditionally been described as a chronic, progressive pathology.
• This case report challenges the traditional definition of maxillary atelectasis.
• Atelectasis developed acutely following endoscopic transnasal transsphenoidal pituitary surgery.
• Combined with other cases of maxillary atelectasis developing acutely the potential pathophysiology of the disease is challenged.

## 1 Introduction

Maxillary atelectasis is a rare condition that describes a reduction in the volume of the maxillary sinus secondary to inward bowing of the antral walls. It was first reported in 1964 by Montgomery [1] and has traditionally been characterised as an acquired condition that follows a chronic, indolent course, leading to the term chronic maxillary atelectasis (CMA). The condition has since been classified into three stages based on gradual and progressive features: stage I is a membranous deformity characterised by lateralisation of the posterior maxillary fontanelle; stage II is a bony deformity with inward bowing of one or more osseous walls; and stage III presents clinically with enophthalmos, hypoglobus and/or midfacial deformity [2]. Silent sinus syndrome (SSS) is a subtype of stage III CMA, characterised by spontaneous enophthalmos, hypoglobus and radiographic evidence of maxillary sinus atelectasis in the absence of sinonasal signs or symptoms. The indolent and often asymptomatic course of disease, combined with its relatively low prevalence and requirement for radiologic diagnosis, makes CMA a relatively uncommon pathology.

It is believed that most patients with maxillary atelectasis remain in a subclinical state for months to years, however few studies have been performed to clarify the specific time course of disease. In the English literature there are only six reported cases of patients with normal initial CT scans and subsequent scans demonstrating maxillary atelectasis over a documented period of time (between three months and twelve months) [[3], [4], [5], [6], [7], [8]].

We present the first reported case of the development of maxillary atelectasis within five months following endoscopic pituitary surgery, managed at a tertiary public Australasian hospital. This case supports the notion that acquired maxillary atelectasis may occur more acutely than previously believed. This case report is reported in line with the SCARE criteria [9].

## 2 Presentation of case

**Fig. 1:**
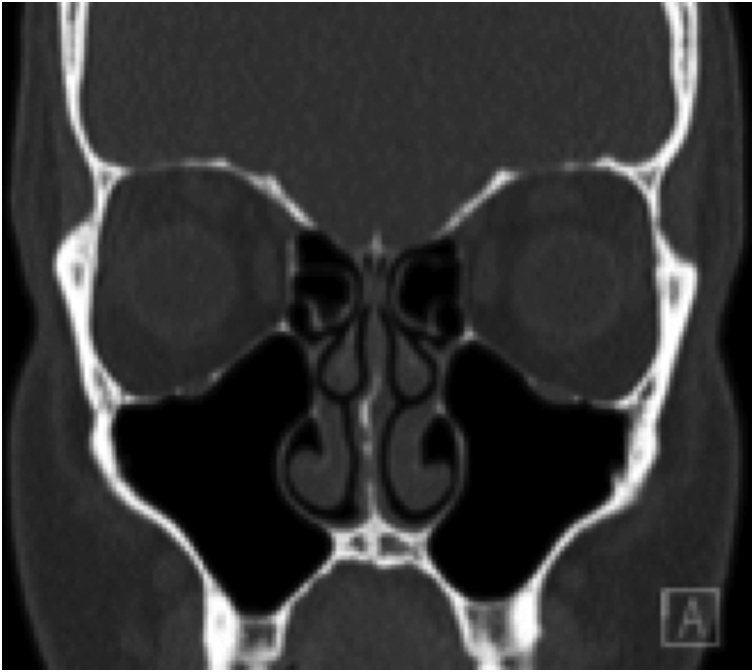
Coronal slice of the paranasal sinuses prior to pituitary surgery demonstrating only a mild right sided nasal septal deviation and no other significant paranasal sinus pathology.

A 29-year-old man presented two months following endoscopic transnasal, transsphenoidal excision of a Rathke’s cleft cyst of the pituitary gland with symptoms of bilateral facial pain and pressure as well as a dull frontal headache. He had no other significant past medical history. The initial diagnosis of a pituitary lesion was made on imaging after he presented with headaches and nasal obstruction. There was a previous history of nasal trauma and rhinoplasty, elsewhere. He was noted to have a persistent right septal deviation and unremarkable sinus anatomy on CT (Fig. 1). The pituitary procedure had been uneventful, employing an endoscopic approach with initial lateralisation of the middle turbinates and partial excision of the superior turbinates to improve access. At the conclusion of the case, the right septal deviation was corrected, a right nasoseptal flap was used to close the sphenoid defect and the middle turbinates were returned to their medial position. The uncinates and antral ostia were otherwise not involved in the procedure. Silastic septal splints were left in-situ for 4 weeks, in view of his right nasoseptal flap.

Two months post-operatively the patient presented complaining of bilateral facial pressure when leaning forward. He was commenced on a trial of medical management directed toward the sinuses, including oral and intranasal steroids, macrolide antibiotics and saline rinses and asked to return with a new CT scan. Despite an initial improvement with medication, his facial pain recurred and he re-presented at five months post-operatively.

**Fig. 2:**
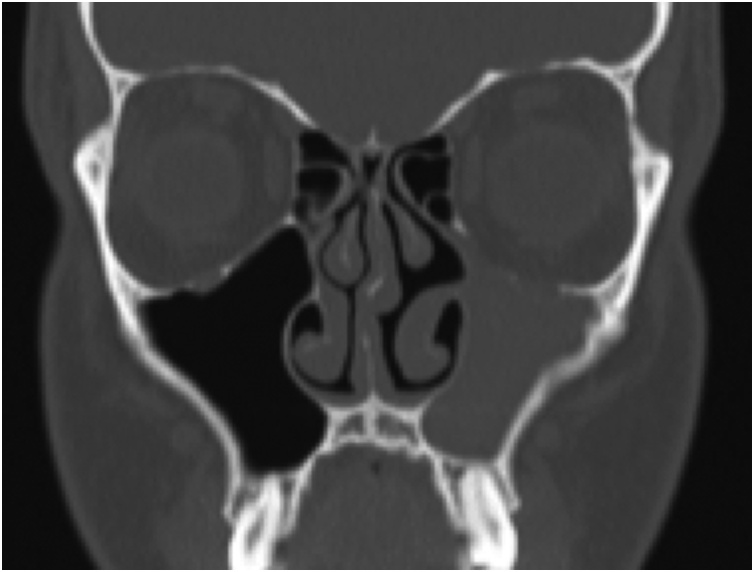
Coronal slice of the paranasal sinuses of the same patient demonstrating interval development of stage 2 maxillary atelectasis following pituitary surgery.

Nasendoscopic examination showed lateralisation of the left uncinate process. A CT scan of the paranasal sinuses demonstrated complete left maxillary opacification and lateralisation of the uncinate process and lateral nasal wall (Fig. 2). The remaining sinuses were unremarkable. These findings were not present on a CT performed 2 weeks prior to the initial pituitary surgery.

The patient proceeded to endoscopic sinus surgery with left uncinectomy and antrostomy, performed by a tertiary fellowship trained rhinologist. Thick mucous was drained from the atelectatic left antrum. The patient was asked to perform regular saline nasal douching until final outpatient review.

At 2 months post-operatively, the patient reported symptom resolution, and nasendoscopic examination demonstrated a widely patent maxillary sinus ostium. A further follow-up appointment at 5 months post-operatively demonstrated no evidence of symptom recurrence.

## 3 Discussion

This case report describes symptoms suggestive of maxillary atelectasis within 2 months following endoscopic pituitary surgery with demonstrable radiological changes over a six month period. This presentation challenges the notion that chronic maxillary atelectasis, as implied by its name, always carries a chronic and indolent course. A small number of previous cases described in the literature that have reported development of maxillary atelectasis within 3–12 months also appear to support this theory [[3], [4], [5], [6], [7], [8]].

The development of spontaneous enophthalmos secondary to maxillary atelectasis following functional endoscopic sinus surgery (FESS) was first reported in 2004 [3]. In that report, the patient developed symptoms three months post-operatively. A pre-operative CT demonstrated a normal left maxillary sinus with symmetrical maxillary sinuses and orbital floors with patent ostia. The repeat CT of the paranasal sinuses demonstrated opacification of the left maxillary sinus with a descending orbital floor and a medially bowing posterior lateral wall. These findings suggest that the patient developed maxillary atelectasis within a three-month period following endoscopic sinus surgery. Subsequent studies have since been published demonstrating acute development of maxillary atelectasis both in the context of recent surgery and occurring de novo (Table 1) [[4], [5], [6], [7], [8]].

**Table 1:** Summary table demonstrating the published literature on rapid development of maxillary atelectasis in the context of both recent sinonasal surgery and spontaneously.

Case	Time to development	Details
**Following previous sinus surgery**		
Wu et al. [3], 2004	3 months	Development of spontaneous enophthalmos with CT demonstrating left maxillary sinus opacification, descending orbital floor and medially bowing lateral wall, following functional endoscopic sinus surgery
Jung and Gray [4], 2012	6 months	Development of left eye enophthalmos following septoplasty and outfracture of the inferior turbinates
Ferri et al. [5], 2012	4 months	Initially diagnosed with hypoplastic and opacified left sided maxillary sinus with depression of the orbital floor and lateralised uncinate process, with a normal right sided maxillary sinus
		Following endoscopic left uncinectomy, antrostomy and orbital floor reconstruction, evidence of RIGHT sided silent sinus syndrome.
**Without previous sinus surgery**		
Eto et al. [6], 1995	12 months	Spontaneous development of right silent sinus syndrome despite MRI prior showing normal maxillary sinus
Elkhatib and House [7], 2017	11 months	Initial left periorbital and retro-orbital pain with nasal obstruction, with no radiological findings on CT and subsequent resolution of symptoms.
		Representation with repeat CT demonstrating left Stage II CMA

The aetiology of maxillary atelectasis is believed to be due to maxillary ostiomeatal complex obstruction resulting in resorption of sinus mucosal gas and the development of negative intra-sinus pressure gradients. Consequently, there is remodelling and demineralisation of the bone, causing subsequent thinning and inward bowing of the maxillary sinus walls. This results in a persistent and progressive reduction in maxillary sinus volume and antral wall collapse [2,10,11]. Although maxillary atelectasis is widely reported in the literature as being a chronic condition, several case reports have documented the development of clinical and radiological signs of maxillary atelectasis within twelve months, thus challenging this traditional description. Restoring ostiomeatal airflow in maxillary atelectasis, typically via an endoscopic uncinectomy and antrostomy is the accepted standard of care and long-term cure of the pathology.

The reports of maxillary atelectasis following surgical intervention indicate that obstruction of the ostiomeatal complex may be iatrogenic in nature. This may be due to instrument trauma intra-operatively or lateralisation of the middle turbinate or uncinate process. The formation of nasal adhesions may also cause obstruction of the ostiomeatal complex [5].

In our case, the left maxillary ostium and uncinate were not directly involved in the initial procedure. It is unclear as to whether the procedure triggered the pathophysiological process leading to maxillary atelectasis. We speculate that lateralisation of the middle turbinate or the use of septal splints may possibly have contributed to ostium obstruction. Our case represents the first report in the literature of maxillary atelectasis occurring a few months following pituitary surgery in a previously normal sinus.

## 4 Conclusion

Maxillary atelectasis is traditionally described as a chronic, progressive entity. We report a case of a patient who developed radiological evidence of left maxillary atelectasis within six months following endoscopic transnasal transsphenoidal pituitary surgery. We therefore challenge the ‘chronic’ nature in traditional descriptions of maxillary atelectasis. Surgeons should be made aware of the potential risk of post-operative maxillary atelectasis when performing any sinonasal surgery, even if the maxillary sinus is not specifically addressed. Further research into this condition is required in order to assess the need to incorporate acute cases into the current classification system of maxillary atelectasis, and to determine what the specific causes of acute atelectasis are.

## Sources of funding

None to declare

## Ethical approval

Ethics approval was obtained from the Western Sydney Local Health District Human Research Ethics Committee (1908-01). The authors assert that all procedures contributing to this work comply with the ethical standards of the relevant national and institutional guidelines on human experimentation and with the Helsinki Declaration of 1975, as revised in 2013.

## Consent

We confirm that informed consent was obtained.

## Author’s contribution

All authors (JH, DR, EW, NS) contributed to study concept and design, data collection, analysis, manuscript preparation and final approval of the manuscript for submission.

## Registration of research studies

Not applicable.

## Guarantor

Dr Eugene Wong, the corresponding author is the guarantor for this study.

## Provenance and peer review

Not commissioned, externally peer-reviewed.

## Declaration of Competing Interest

None to declare.

## References

[1] Montgomery W. (1964). Mucoceles of the maxillary sinus causing enophthalmos. Eye Ear Nose Throat Mon..

[2] Kass E.S., Salman S., Weber A.L., Rubin P.A., Montgomery W.W. (1997). Chronic maxillary atelectasis. Ann. Otol. Rhinol. Laryngol..

[3] Wu C.-L., Hsu M.-C., Liu C.-M. (2004). A rare complication of functional endoscopic sinus surgery: maxillary atelectasis-induced spontaneous enophthalmos. Am. J. Rhinol..

[4] Jung D., Gray S.T. (2012). Silent sinus syndrome after lateral fracture of the inferior turbinate. Otolaryngol. Head. Neck Surg..

[5] Eloy J.A., Jacobson A.S., Elahi E., Shohet M.R. (2006). Enophthalmos as a complication of rhinoplasty. Laryngoscope.

[6] Ferri A., Ferri T., Sesenna E. (2012). Bilateral silent sinus syndrome: case report and surgical solution. J. Oral Maxillofac. Surg..

[7] Eto R.T., House J.M. (1995). Enophthalmos, a sequela of maxillary sinusitis. Am. J. Neuroradiol..

[8] Elkhatib A., McMullen K., Hachem R.A., Carrau R.L., Mastros N. (2017). Rapidly progressive maxillary atelectasis. J. Craniofac. Surg..

[9] Agha R.A., Borrelli M.R., Farwana R., Koshy K., Fowler A., Orgill D.P., For the SCARE Group (2018). The SCARE 2018 statement: updating consensus Surgical CAse Report guidelines. Int. J. Surg..

[10] Numa W.A., Desai U., Gold D.R., Heher K.L., Annino D.J. (2005). Silent sinus syndrome: a case presentation and comprehensive review of all 84 reported cases. Ann. Otol. Rhinol. Laryngol..

[11] Kass E.S., Salman S., Montgomery W.W. (1996). Manometric study of complete ostial occlusion in chronic maxillary atelectasis. Laryngoscope.

